# Advances in the treatment of autoimmune nodopathy: based on treatment strategies of CIDP

**DOI:** 10.3389/fimmu.2026.1735745

**Published:** 2026-03-11

**Authors:** Zhi-Dan Pang, Hui Sun, Xiao-Jing Wei, Chang-Pu Nie, Xue-Fan Yu

**Affiliations:** Department of Neurology and Neuroscience Center, The First Hospital of Jilin University, Changchun, China

**Keywords:** autoantibody, autoimmune nodopathy, CIDP, immunotherapy, rituximab

## Abstract

Chronic Inflammatory Demyelinating Polyneuropathy (CIDP) is an autoimmune peripheral neuropathy primarily characterized by macrophage-mediated demyelination. Studies have identified that some patients possess autoantibodies against contactin-1 (CNTN1), neurofascin-155 (NF155), contactin-associated protein 1(Caspr1), and neurofascin-186/140 (NF186/140). Based on the unique pathogenesis and pathological features, the 2021 European Academy of Neurology/Peripheral Nerve Society (EAN/PNS) guidelines have categorized these patients separately, defining them as “Autoimmune Nodopathy (AN)”. The standard first-line treatments for CIDP include corticosteroids, intravenous immunoglobulin (IVIG), and plasma exchange. If these treatments are ineffective or poorly tolerated, it can be replaced with immunosuppressants or used in combination. Emerging therapeutic strategies are also being explored, among which subcutaneous injection of efgartigimod, a recently approved drug, is gradually accumulating clinical application value. The treatment strategy for AN differs from that of CIDP: Rituximab is currently regarded as the preferred option for treating AN, with corticosteroids being effective for some patients. Plasma exchange can be utilized for severe cases, while IVIG is largely ineffective for most patients with AN. Due to the low incidence of AN and the limited clinical evidence available, its treatment strategies still require large-scale clinical trials for validation. This article systematically reviews the treatment advancements for CIDP and focuses on the unique treatment strategies for AN.

## 1 Introduction

Chronic Inflammatory Demyelinating Polyneuropathy (CIDP) is an autoimmune neuropathy that primarily affects peripheral nerves and nerve roots. It is characterized by symmetric motor and sensory disturbances in the limbs, which may be accompanied by ataxia and tremors, with a chronic progression lasting more than eight weeks (1). Although the pathogenesis of CIDP remains unclear, macrophage-mediated demyelination is widely accepted as a key pathophysiological mechanism. The pathological features include the destruction of myelinated fibers by macrophages, leading to demyelination around the basement membrane and the formation of a distinctive “onion bulb” structure (2). Traditional treatment methods for CIDP mainly include corticosteroids, plasma exchange (PE), and intravenous immunoglobulin (IVIG). The subcutaneous injection of efgartigimod, an approved targeted therapy, is also gaining increasing attention, with more clinical trials underway to explore emerging treatment methods.

**Figure 1:**
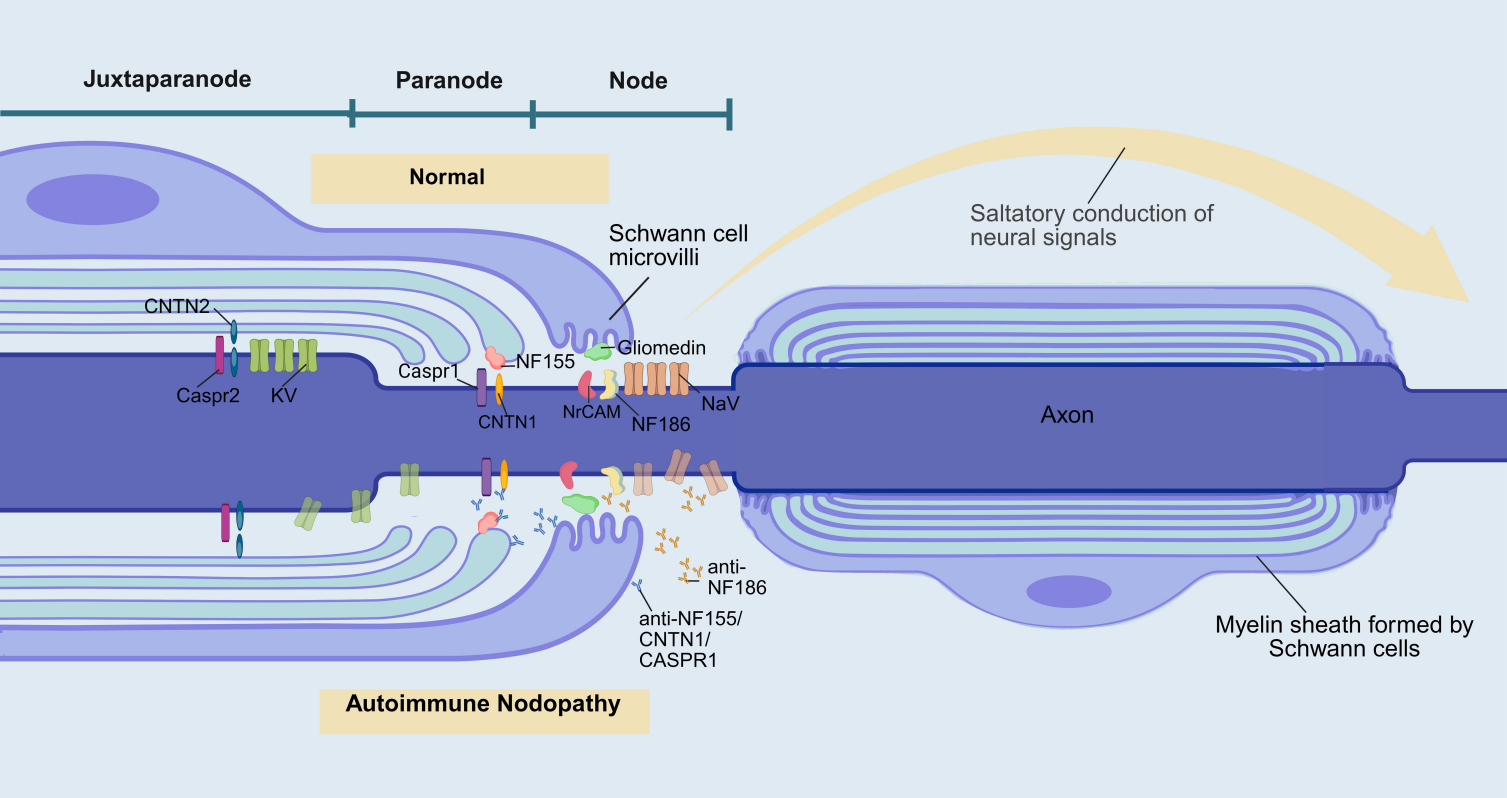
Pathogenesis of autoimmune nodopathy and functions of proteins in the nodal/paranodal region. (Created in BioRender) The Ranvier node is a small, short interval on the axon that is not covered by myelin, which is crucial for the saltatory conduction of nerve signals and consists of three parts: the node, the paranode, and the juxtaparanode region. NF186/140 maintains the stability of the Ranvier node structure and promotes the aggregation of Na+ channels by binding to NrCAM and Gliomedin on the microvilli of Schwann cells. The complex formed by NF155 with CNTN1 and Caspr1 separates the Na+ channels at the node and the K+ channels at the juxtaparanode, creating a barrier against the lateral diffusion of nodal channels and anchoring the myelin sheath to the axonal membrane. When antibodies against the nodal/paranodal region bind to their target proteins, the stability of these structures is disrupted, ultimately leading to demyelinating lesions in peripheral nerves: Anti-NF186 antibodies directly disrupt the structure of the nodal complex, leading to a disordered distribution of sodium channels and resulting in conduction block. NF155 antibodies cause remodeling of the juxtaparanodal region, while CASPR1/CNTN1 antibodies directly damage the protein complex in the juxtaparanodal region. Together, they disrupt the anchoring between Schwann cells and axons, leading to demyelination, which in turn causes potassium channel diffusion and ectopia (156, 157).. NaV voltage-gated sodium channel, KV voltage-gated potassium channel, NrCAM neuronal cell adhesion molecule, NF neurofascin, CNTN contactin, Caspr contactin-associated protein.

Current research indicates that various autoantibodies can lead to different pathogenic mechanisms and pathological features compared to typical CIDP, particularly autoantibodies targeting contactin-1 (CNTN1), neurofascin-155 (NF155), contactin-associated protein 1 (Caspr1), and neurofascin-186/140 (NF186/140). These autoantibodies disrupt the binding of the myelin terminal loops to the axolemma, resulting in significant conduction defects (the specific pathogenic mechanisms are illustrated in **Figure 1**). Therefore, the 2021 guidelines from the European Academy of Neurology/Peripheral Nerve Society (EAN/PNS) (1) proposed distinguishing this patient category from CIDP, naming it” Autoimmune Nodopathy (AN)”.They recommended testing for nodal and paranodal antibodies in patients who meet the CIDP diagnostic criteria but present with atypical clinical features.

The clinical features of AN are generally similar to those of CIDP, but there are also some unique manifestations such as tremors, cranial nerve involvement, and sensory ataxia, with cerebrospinal fluid protein levels typically significantly elevated (3). Additionally, conduction block, distal motor latency, and F-wave abnormalities on nerve electrophysiology may be more pronounced (4).In terms of pathological characteristics, it lacks the notable macrophage-mediated demyelination and “onion bulb” structures seen in CIDP (5),instead exhibiting structural damage in the Ranvier nodes and demyelination in the paranodal regions. Most patients with AN show poor or ineffective responses to IVIG (6), while rituximab has shown better efficacy (7).

Early diagnosis and treatment, as well as the exploration of more effective therapeutic methods, are of significant importance for CIDP. As a newly defined disease, there is currently limited systematic research on the treatment of AN. This paper will summarize the advancements in the treatment of CIDP and focus on the unique treatment characteristics and latest therapeutic developments of AN, aiming to provide a reference for the treatment of the disease.

## 2 The treatment of CIDP

**Figure 2:**
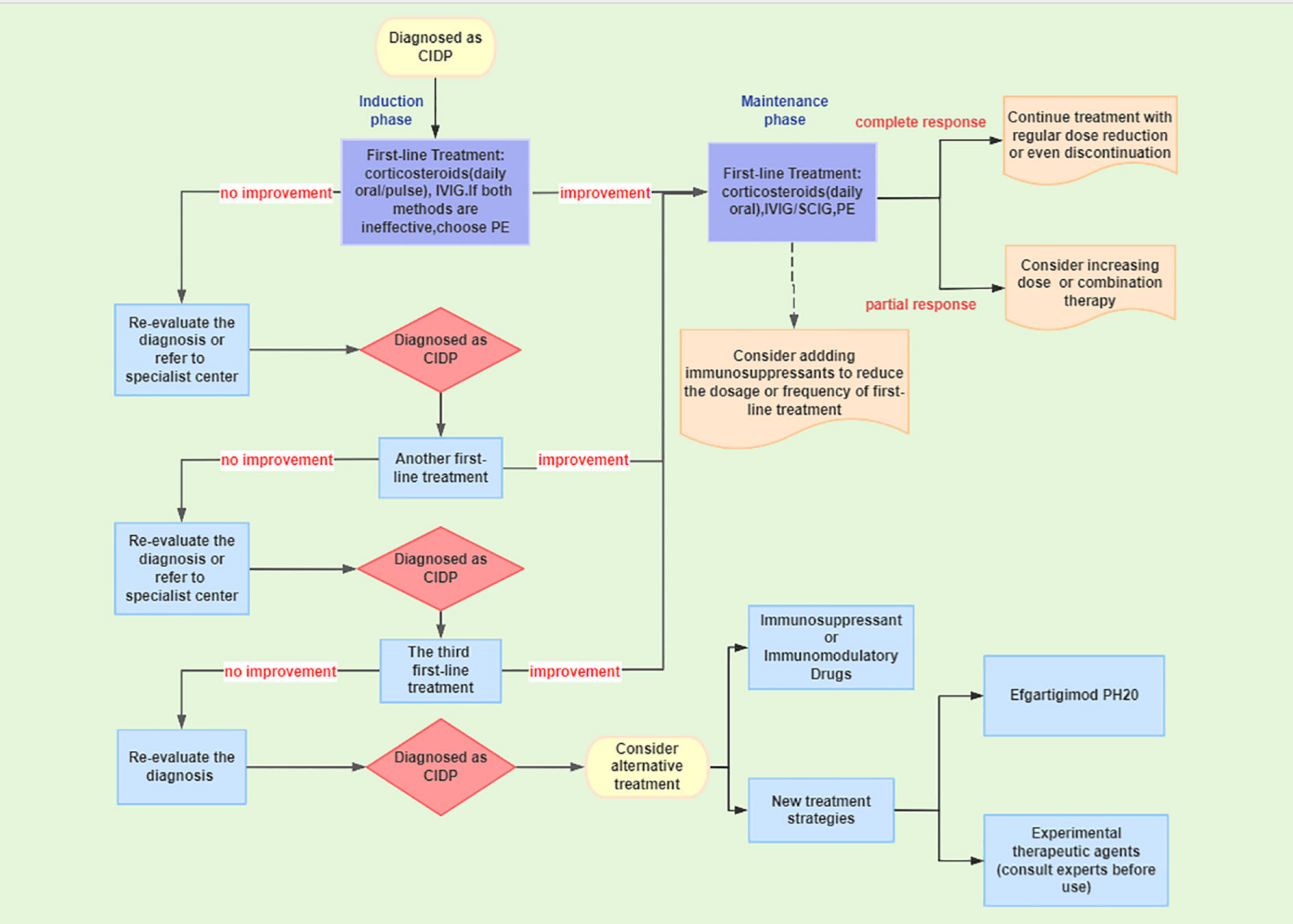
Treatment pathway for CIDP.

The treatment of CIDP follows a stepwise strategy (**Figure 2**): the first-line treatments include IVIG, corticosteroids, or plasma exchange to rapidly induce remission, with approximately 80% of CIDP patients responding effectively to first-line treatment (8). Among these, IVIG and corticosteroids are the preferred choices. If both are ineffective, PE should be considered. If the efficacy of first-line treatment is poor or cannot be tolerated long-term, immunosuppressants may be switched or combined as a salvage therapy or adjunct treatment to achieve maintenance therapy. Many emerging treatment methods have shown therapeutic value, particularly efgartigimod, which is suitable for patients who have insufficient response to existing first-line treatments, are intolerant, or require convenient alternative options (1).

### 2.1 First-line treatment

#### 2.1.1 Glucocorticoids

Glucocorticoids can alleviate neuroinflammation and demyelination, thereby improving patient symptoms by inhibiting immune cell activity and reducing the release of pro-inflammatory cytokines. As early as 1958, Austin (9) observed significant effects of glucocorticoids in patients with CIDP who experienced relapses after cessation of treatment. Since then, research on corticosteroid treatment for CIDP has increased, and in the 2008 EFNS guidelines (10), glucocorticoids were explicitly recommended as a first-line treatment method for widespread application worldwide. Currently, glucocorticoids are still used as a first-line treatment in clinical practice. The administration methods of corticosteroids mainly include oral prednisone/prednisolone, pulsed dexamethasone, and intravenous methylprednisolone. But the optimal corticosteroid regimen is still unclear. Some studies have indicated that high-dose intermittent intravenous pulse methylprednisolone (11) and high-dose pulse dexamethasone (12) are more effective than long-term oral steroid therapy and can significantly reduce steroid-related adverse reactions. Currently, a common medication regimen (1) involves daily oral prednisolone 60mg, maintained for 4 weeks before gradually tapering. Alternatively, daily pulse oral dexamethasone 40mg can be administered for 4 consecutive days every 4 weeks, sustained for 6 months. For more severe cases, intravenous methylprednisolone 500mg-1000mg/d can be administered for 3–5 days before gradually tapering or switching to oral prednisone (13). Adverse reactions such as osteoporosis, electrolyte imbalance, and hyperglycemia are noteworthy in the application of glucocorticoids.

#### 2.1.2 Immunoglobulins

Immunoglobulins have the functions of neutralizing pathogenic antibodies, inhibiting the production of pathogenic antibodies, blocking local cell Fc receptor responses, and complement suppression, thereby inhibiting autoimmune responses (14, 15).In 1994, a crossover study by Dyck compared IVIG at a dose of 0.4 g/kg per week with plasma exchange, demonstrating that IVIG treatment is beneficial for CIDP (16). Subsequently, a large, randomized, placebo-controlled, crossover trial (ICE) further confirmed the short-term and long-term efficacy of IVIG (17). Since then, IVIG has been widely used worldwide, becoming the first-line treatment for CIDP due to its rapid efficacy and low incidence of side effects. Common side effects include headache, fever, hypertension, while severe adverse reactions such as thromboembolism, renal failure, and anaphylactic shock are relatively rare (18). Screening for risk factors such as vascular diseases, renal insufficiency, and IgA deficiency may reduce the occurrence of serious complications.

Long-term IVIG maintenance therapy may pose a risk of overtreatment (19), thus it is important to determine the appropriate dosage and frequency for maintenance therapy. The EAN/PNS recommends a maintenance dose of IVIG at 1 g/kg every 3 weeks (13), but there is currently no unified standard for the optimal maintenance dose and treatment interval. A randomized placebo-controlled crossover trial compared two IVIG maintenance treatment approaches: low-dose with high frequency and high-dose with low frequency. No significant differences in outcomes and side effects were observed between the two approaches (20). A Phase III clinical study (CTR20250745) is currently underway, which observes the efficacy of maintenance doses of 1.0g/kg or 2.0g/kg following a loading dose of 2.0g/kg, potentially providing further clinical evidence for the optimal maintenance dosage of IVIG in the treatment of CIDP.

Subcutaneous immunoglobulin (SCIG) is a safe and effective maintenance therapy for CIDP. Compared to IVIG, SCIG has fewer systemic adverse reactions (21) and can be administered at home by patients or caregivers, facilitating home and self-management. However, it typically requires more frequent infusions, such as once or twice a week. A 12-week RCT conducted in Denmark involving 30 CIDP patients who had previously received IVIG treatment showed significant improvements in multiple metrics and disability in the SCIG treatment group compared to the placebo group (22). This indicates that SCIG treatment for CIDP is feasible, safe, and effective, and it holds promise as an alternative to IVIG. A crossover study targeting treatment-naive CIDP patients revealed similar results for short-term SCIG and IVIG treatment regarding motor improvement, although IVIG showed the greatest improvement early on, suggesting the potential for SCIG treatment in newly diagnosed CIDP patients (23). Subsequently, a large international RCT involving 172 subjects from 69 centers further confirmed that both low-dose (0.2 g/kg weekly) and high-dose (0.4 g/kg weekly) SCIG were well tolerated and effectively reduced the risk of relapse, demonstrating the therapeutic effect of SCIG during the maintenance phase of CIDP (24). Currently, SCIG is only recommended for maintenance therapy in CIDP, and an ongoing trial (NCT04589299) aims to compare the effects of SCIG and IVIG in 60 treatment-naive CIDP patients.

Hyaluronidase can increase the permeability of subcutaneous tissue to immunoglobulins, facilitating the dispersion and absorption of SCIG into the bloodstream, thereby effectively addressing the issue of the maximum volume that can be infused into the subcutaneous space (25). Consequently, compared to traditional SCIG, hyaluronidase-facilitated SCIG (fSCIg) can reduce the frequency of infusions, the duration of infusions, and the number of needle sticks required. A large randomized controlled trial (ADVANCE-CIDP 1) involving 132 subjects receiving 10% hyaluronidase-facilitated SCIG has demonstrated its efficacy in reducing relapse rates by over 20% compared to placebo (26). This indicates that this medication can prevent the recurrence of neuromuscular disabilities and the deterioration of function in CIDP. Based on these results, fSCIg 10% has been approved in the United States for the maintenance treatment of adult CIDP and in Europe for adults and children with CIDP who are stable after IVIG treatment. Its long-term extension study (ADVANCE-CIDP 3) also indicates that fSCIg has long-term safety and tolerability, with a low relapse rate, supporting its use as maintenance therapy for CIDP (27).

#### 2.1.3 Plasma exchange

Plasma exchange exerts immunomodulatory effects by rapidly removing pathogenic autoantibodies, antigen-antibody complexes, complement, and cytokines from the blood by separating and replacing the patient’s plasma (28). Therefore, PE can significantly improve clinical impairment and disability in patients with CIDP, particularly in severe cases and those with recurrent diseases (29). In 1996, Hahn et al. (30) performed a crossover trial that involved ten sessions of PE over four weeks, finding that 80% of patients experienced significant improvements in nerve function after PE treatment. However, 66% of patients relapsed within 7 to 14 days after discontinuing PE. Subsequent smaller trials indicated that 48% to 81% of patients with CIDP achieved short-term improvements following PE treatment, but a rapid deterioration could occur after the completion of treatment (31, 32). These findings limit the long-term clinical application of PE, which is currently used primarily for patients with CIDP who are unresponsive or intolerant to intravenous immunoglobulin and corticosteroids. Adverse events related to plasma exchange are rare and generally mild (33). The most common adverse events include access issues, hypocalcemia, hypotension, allergic reactions, and bleeding tendencies.

Immunoadsorption (IA) is considered a potential alternative treatment for plasma exchange, with increasing research on its application in CIDP in recent years. Compared to plasma exchange, immunoadsorption can selectively remove immunoglobulins without the need for replacement fluids, thereby reducing the incidence of complications such as bleeding and allergic reactions. A prospective randomized trial comparing the use of immunoadsorption (IA) or PE in 20 patients with CIDP indicated that the clinical improvement rates and side effects of both treatments were similar (34). In another prospective study involving 17 patients (35), 85.7% of patients did not experience any related disease progression under routine IA treatment, demonstrating its potential for long-term treatment of CIDP. In a recent retrospective study (36), IA and PE demonstrated comparable efficacy and safety in patients with a longer disease course and those who underwent immunomodulatory pretreatment. An ongoing randomized controlled trial (NCT04881682) aims to evaluate the safety and efficacy of immunoadsorption compared to immunoglobulin in steroid-resistant CIDP. However, both PE and IA cannot prevent the continuous production of autoimmune antibodies in patients, necessitating periodic treatment.

### 2.2 Existing immunosuppressant and immunomodulatory drugs

Research indicates that approximately 25% of CIDP patients are refractory, meaning they do not respond to any of the three first-line treatments: corticosteroids, IVIG, or plasmapheresis (37, 38). For patients who are insufficiently responsive to first-line treatments, cannot tolerate them, or find them inconvenient, some immunosuppressants or immunomodulators may be selected (1). Additionally, during the maintenance treatment phase, these medications can be used in combination to assist in reducing the dosage of first-line treatments, thereby minimizing adverse reactions. Currently, rituximab (RTX) and cyclophosphamide (CYC) are the primary treatment options for refractory CIDP (39).

#### 2.2.1 Cyclophosphamide

Cyclophosphamide is an alkylating agent that exerts immunosuppressive effects by cross-linking DNA and RNA chains, thereby inhibiting protein synthesis. It has currently been utilized in the treatment of immune-mediated neuropathies that are unresponsive to first-line therapies. A study conducted by Good et al. (40) involving 15 subjects with refractory conditions to three different first-line treatments demonstrated that monthly cyclophosphamide pulse therapy, in conjunction with high-dose corticosteroids, resulted in improvements for all patients within an average of 3.3 months, achieving a complete remission rate of 73.3%. The improvements were sustained for up to 9 years, suggesting that cyclophosphamide may facilitate long-term clinical remission. Subsequent reports from smaller series have shown similar results (41). Therefore, cyclophosphamide is currently frequently employed as the preferred immunosuppressant for severe refractory CIDP in many centers.

According to case series reports, cyclophosphamide pulse therapy may be preferable to low-dose or oral administration. It is recommended to administer via intravenous route at a dosage of 1 g/m^2^ once a month for a maximum duration of 6 months, unless significant early improvement occurs. Concurrent use of high-dose corticosteroids is routinely recommended, although some studies have indicated that related corticosteroid treatment does not provide additional benefits (42). It is noteworthy that repeated cyclophosphamide treatment requires careful consideration of the risk of side effects. CYC has significant immunosuppressive effects and notable cytotoxic properties, commonly leading to adverse reactions such as bone marrow suppression, reproductive system damage, hemorrhagic cystitis, and pulmonary fibrosis (42). For patients who can tolerate CYC and do not exhibit severe adverse events, individualized treatment dosages of CYC can be gradually explored.

#### 2.2.2 Rituximab and other monoclonal antibodies

Rituximab is a monoclonal antibody against CD20 that binds to the CD20 molecule on the surface of B cells, leading to B cell depletion, thereby reducing the production of pathogenic antibodies and the activation of other immune cells (43). In recent years, the positive therapeutic effects of rituximab on CIDP have been confirmed, particularly in refractory cases. Case series studies have shown the efficacy of rituximab in 63% of CIDP patients, 48% of patients with anti-MAG neuropathy, and 96% of patients with AN (44). In 2011, Benedetti et al. (45) reported that 69% (9/13) of patients with refractory CIDP showed improvement in symptoms following RTX treatment, with effects lasting from 2 to 12 months. In 2020, Muley et al. (46) reported significant improvements in disability scores and limb function in 11 patients with refractory CIDP after RTX treatment.

RTX appears to play a role in reducing the frequency and cost of traditional plasma exchange and IVIG infusions. However, an Italian randomized, double-blind, placebo-controlled trial randomly assigned 37 CIDP patients receiving IVIG treatment to either the rituximab or placebo group, with no significant differences observed between the two groups. This suggests that rituximab does not have a significant effect in preventing clinical deterioration after CIDP patients discontinue immunoglobulin treatment (47).An ongoing clinical trial (NCT06714838) is investigating the use of RTX treatment in both untreated and Ig-dependent patients to further explore the long-term significance of incorporating RTX therapy into Ig treatment.

The common dosing regimen for rituximab in CIDP is either 1g per infusion administered once every two weeks for a total of two doses or 375 mg/m^2^ given weekly for four consecutive weeks. Re-treatment is permitted after 4 to 6 months. However, it is not necessary for patients who have achieved remission or are close to complete remission. Nevertheless, this high-dose rituximab regimen may lead to extremely low B cell counts, with an immunocompromised state persisting for at least 6 months before returning to normal (48), significantly increasing the risk of infections. Therefore, close monitoring of B cell counts is required before and after treatment to ensure safety.

In addition to the widely used RTX, there are currently many monoclonal antibodies clinically used for the treatment of CIDP. Ocrelizumab, a more humanized anti-CD20 antibody than RTX, is believed to have lower immunogenicity and better tolerability. Currently, only case reports have confirmed its therapeutic value in CIDP (49, 50). Ofatumumab is a fully humanized anti-CD20 monoclonal antibody and is the only drug that can be administered subcutaneously (51). Obinutuzumab, a rituximab analogue, exhibits stronger ADCC effects and direct cytotoxicity compared to RTX (52). It can be applied in cases of rituximab resistance but may lead to increased toxicity and higher costs (53). In 2015, Vallat et al. (54) reported that three patients with refractory CIDP showed improvement or stabilization of symptoms after using natalizumab. Alemtuzumab, a humanized anti-CD52 monoclonal antibody, exerts immunosuppressive effects by inducing permanent apoptosis of lymphocytes (55), demonstrating good efficacy in relapsing-remitting MS and chronic B-cell leukemia (56).In March 2010, Marsh et al. (57) reported on seven patients with CIDP who were treated with alemtuzumab, of which four showed symptom improvement and two achieved complete remission. The clinical trial for alemtuzumab in CIDP was withdrawn in 2017 (NCT 01757574), with no further trials planned. Given the concerns regarding its safety, the use of alemtuzumab may be limited. Currently, the majority of monoclonal antibodies used in the treatment of CIDP lack large-sample evidence-based support, and their efficacy remains uncertain. For CIDP patients who are unresponsive to first-line treatment, the application of these therapies may be considered.

#### 2.2.3 Other immunosuppressants

Azathioprine or mycophenolate mofetil are commonly used in clinical practice to facilitate steroid reduction. Azathioprine is a broad-spectrum immunosuppressant that exerts its effects by inhibiting the DNA synthesis, proliferation of immune cells, and the innate immune response. In a controlled trial by Dyck et al. comparing prednisone with prednisone combined with azathioprine for the treatment of CIDP, no therapeutic effect of azathioprine was found (58). A retrospective study published in Italy in 2011 found that azathioprine was effective in 27% of patients (59). However, this discrepancy may be attributed to the underlying treatment. Nevertheless, azathioprine is still widely regarded as a first-line immunosuppressant for the treatment of CIDP in some low-income countries, possibly due to its effectiveness in other autoimmune diseases and relatively mild side effects (60). Mycophenolate mofetil (MMF) specifically inhibits the activity of inosine monophosphate dehydrogenase in the purine *de novo* synthesis pathway in lymphocytes, thereby suppressing lymphocyte proliferation. Research indicates that it can alleviate symptoms in patients and reduce the dosage of conventional medications (61). A recent retrospective case-control study (62) has demonstrated the practical significance of early addition of MMF in refractory CIDP.

Currently, most studies on the efficacy of immunosuppressants have a common limitation of small sample sizes and a lack of clear evidence for effectiveness. As a result, there is no definitive consensus on the application of these therapies in the treatment of CIDP. Randomized controlled trials (RCTs) evaluating the efficacy of interferon β-1a (63), methotrexate (64), and fingolimod (65) for CIDP have not indicated significant improvements. Additionally, some case reports or small studies (66) have reported the effectiveness of etanercept, interferon-α, fludarabine, and tacrolimus (67), although these are not commonly used in clinical practice (68).

### 2.3 Emerging treatment strategies

**Figure 3:**
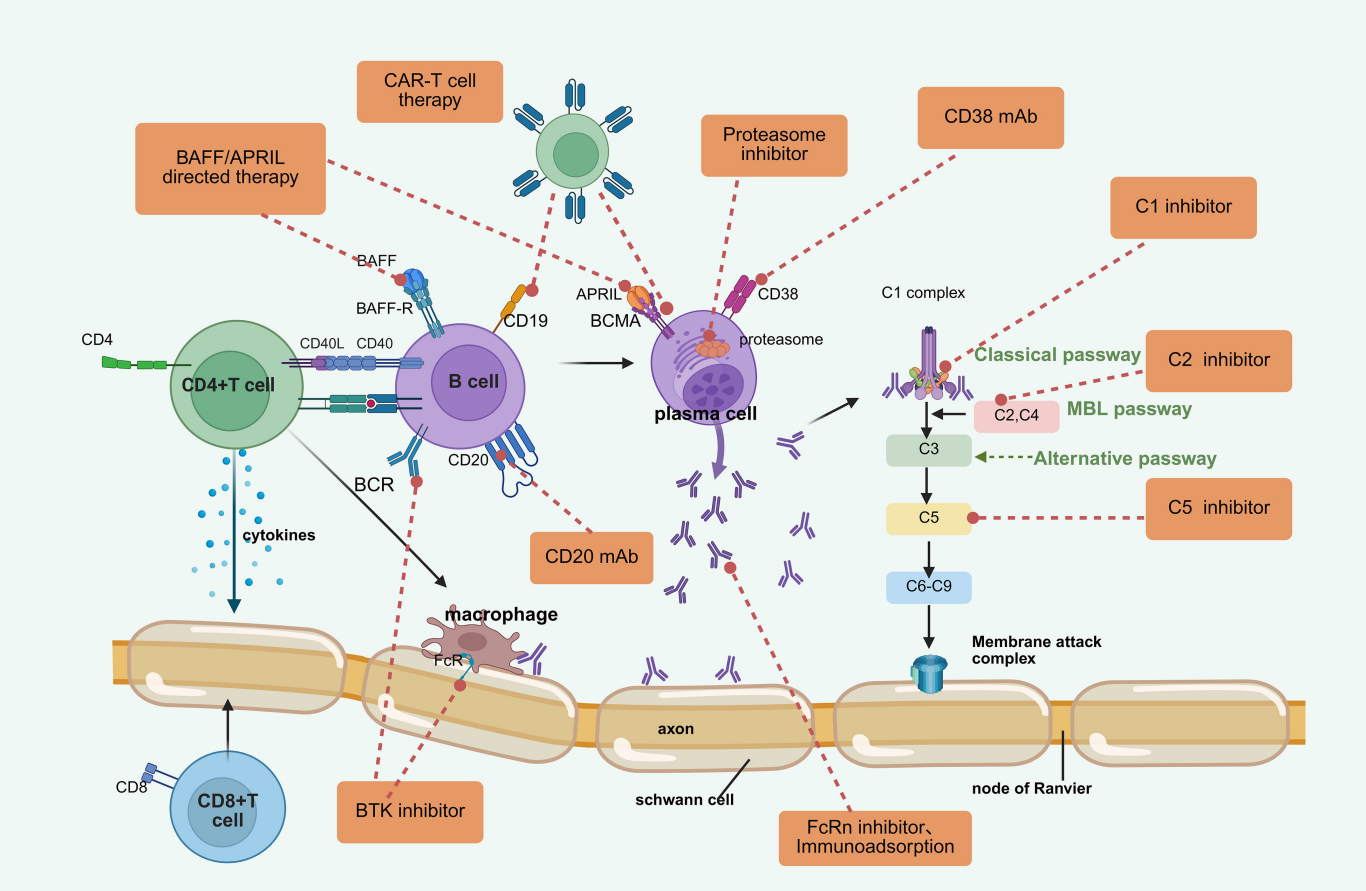
Emerging therapeutic methods and targets for CIDP. (Created in BioRender). BTK bruton tyrosine kinase, mAb monoclonal antibody, BCMA B-cell maturation antigen, BAFF B-cell-activating factor of the TNF family, APRIL a proliferation-inducing ligand, FcR Fc receptor, CAR-T chimeric antigen receptor T-cell.

As the immunopathological mechanisms of CIDP are explored, many targeted drugs and therapeutic approaches have shown therapeutic potential. Although first-line treatments are widely applied, there are still limitations such as poor efficacy in some patients, significant adverse reactions, and inconvenient administration. Emerging treatment strategies aim to address these shortcomings. Current research strategies include reducing the proliferation and activity of B cells and plasma cells, eliminating autoantibodies, and inhibiting complement system activation. Moreover, using HSCT to rebuild the immune system is also a promising treatment option for refractory CIDP. These new treatment strategies represent a new direction for CIDP therapy, with some drugs already approved or in clinical trial stages. **Figure 3** summarizes the mechanisms and targets of these emerging therapeutic methods.

#### 2.3.1 Proteasome inhibitor - bortezomib

A novel treatment increasingly used for refractory CIDP is bortezomib, a reversible proteasome inhibitor that disrupts multiple downstream signaling pathways in the cellular and bone marrow microenvironment, and is considered a promising therapeutic approach for POEMS syndrome (69, 70). In a case series treating 10 patients with refractory CIDP (37), bortezomib stabilized 6 cases in the short term and improved clinical and electrophysiological parameters in 4 cases after one year, with no reported related side effects. A retrospective cohort study indicated that bortezomib is effective in treating refractory chronic immune-mediated sensory-motor neuropathy (CIN) and suggested its use as a treatment option for patients refractory to rituximab (71). However, attention should be paid to the dose-dependent peripheral neurotoxic side effects, which are typically accompanied by weakness and painful sensory neuropathy.

#### 2.3.2 FcRn blockers

The neonatal Fc receptor (FcRn) is a class I MHC molecule that binds and recycles IgG, preventing its degradation and significantly extending its half-life in the body. However, in autoimmune diseases, this function also protects pathogenic autoantibodies. FcRn blockers can selectively inhibit FcRn, thereby rapidly clearing antibodies from circulation, offering a potential therapeutic approach for CIDP (72). Existing FcRn antagonists include efgartigimod, rozanolizumab, nipocalimab, and batoclimab. These medications offer convenience over IVIG through subcutaneous self-administration at low doses and do not significantly affect the levels of other immunoglobulins (73). Therefore, FcRn antagonists may achieve optimal efficacy while avoiding adverse reactions. However, the long-term safety of these drugs remains unclear.

Efgartigimod is a humanized IgG1 antibody fragment that blocks FcRn by competitively binding to it, leading to increased degradation of IgG and a rapid reduction in circulating IgG levels, while not affecting other immunoglobulins and albumin such as IgM and IgA (74). The first human study conducted in 2018 with 62 healthy volunteers demonstrated that efgartigimod can reduce all IgG subtypes, with a single dose resulting in a 50% reduction in serum IgG levels, while repeated dosing can achieve up to a 75% reduction (75). Efgartigimod was approved in the United States in 2021 for the treatment of adult acetylcholine receptor antibody-positive generalized myasthenia gravis. A recent case series from China (76) reported on five patients who received intravenous efgartigimod, all of whom exhibited a decrease in IgG levels, with an average reduction of 43%. This suggests the efficacy of efgartigimod in the clinical management of CIDP patients. The results regarding the redistribution of T/B cell subpopulations also indicate a potential immunomodulatory mechanism of agomelatine.

In recent years, the subcutaneous formulation of Efgartigimod PH20 has emerged as a treatment option for CIDP due to its convenience and reduced infusion-related adverse reactions. In the ADHERE study (77), 27.9% of participants receiving subcutaneous efgartigimod PH20 experienced a relapse, compared to a relapse rate of 53.6% in the placebo group. This indicates the efficacy of subcutaneous efgartigimod PH20 in reducing the risk of relapse in CIDP patients who respond to treatment. Based on these results, in June 2024, Efgartigimod PH20 was approved by the FDA in the United States for the treatment of CIDP, and one year later, it also received approval from the European Medicines Agency (EMA). The ADHERE+ study (NCT04280718), which assesses the long-term efficacy and safety of the subcutaneous formulation of efgartigimod may provide evidence for its treatment and long-term management of CIDP. Meanwhile, an ongoing Phase 4 clinical study (NCT06637072) aims to evaluate the adaptability of CIDP patients following IVIG treatment with efgartigimod.

Rozanolixizumab is a humanized FcRn IgG4 monoclonal antibody that effectively reduces IgG1 to IgG4 subtypes (78), demonstrating efficacy in ITP and MG (79). A randomized, double-blind, placebo-controlled small Phase II study (80) assigned CIDP patients receiving subcutaneous or intravenous immunoglobulin maintenance therapy to either the Rozanolixizumab group or the placebo group. No significant differences in efficacy were observed between the two groups. However, IgG levels were significantly reduced, with good tolerance and safety. Although drug-specific effects cannot be excluded, the heterogeneity and lower activity of the disease in the selected patients, along with the high stability of the placebo, may be important factors contributing to this outcome. Nipocalimab and Batoclimab are also promising humanized FcRn monoclonal antibodies (81), proving to be safe and effective in MG (82). Currently, a multicenter Phase II/III randomized double-blind trial for Nipocalimab (NCT05327114) and a Phase 2b quadruple-blind trial for Batoclimab (NCT05581199) are underway to evaluate their efficacy and safety for CIDP.

#### 2.3.3 Chimeric antigen receptor T-cell therapy

Chimeric antigen receptor T-cell (CAR-T) therapy involves the isolation and purification of T cells from the patient’s blood, followed by genetic engineering to equip these cells with chimeric antigen receptors (CAR) that recognize target cells. The amplified CAR-T cells are then reinfused into the patient, allowing them to specifically target and proliferate against the target cells, exerting cytotoxic effects. This therapy has made significant breakthroughs in the treatment of refractory hematological malignancies (83), and has also demonstrated good efficacy and tolerance in autoimmune diseases such as systemic lupus erythematosus (SLE).Common complications associated with CAR-T therapy include Immune Effector Cell-Associated Neurotoxicity Syndrome (ICANS) and Cytokine Release Syndrome (CRS),with an incidence of approximately 40-80% in the treatment of malignant tumors (84), whereas in the treatment of autoimmune diseases, the incidence is lower and mostly mild (85).

In 2024, Zhang et al. (86) reported the first successful case of a patient with refractory CIDP treated with dual-target CAR-T cells (BCMA-CD19). This dual-target treatment aims to simultaneously eliminate B cells and plasma cells while reducing the production of autoantibodies, thereby alleviating nerve damage. The patient showed a nearly normal recovery of muscle strength and significant improvement in mobility, with no relapse within one year. An ongoing study on eight types of recurrent/refractory antibody-related inflammatory neuropathies (NCT04561557) reported that two patients with highly recurrent refractory CIDP achieved drug-free remission within six months after receiving anti-BCMA CAR-T cell therapy, with no serious adverse events occurring (87). This demonstrates the safety and potential of this therapy for treating refractory CIDP.A recent report on the application of anti-CD19 CAR-T therapy has also shown promising results (88).Current research on CAR-T therapy primarily consists of case reports, and large-scale clinical trials are still needed to validate its efficacy and safety.

#### 2.3.4 Hematopoietic stem cell transplantation

Hematopoietic stem cell transplantation (HSCT) eliminates self-pathogenic immune cells through high-dose chemotherapy and radiotherapy, followed by the reinfusion of autologous hematopoietic stem cells. This process reconstructs a new immune system that tolerates autoimmunity, thereby alleviating clinical symptoms and promoting patient recovery (89). HSCT has been widely applied in the treatment of refractory autoimmune diseases, with evidence suggesting its potential efficacy in treating CIDP, particularly in severe and refractory cases.

In 2002, Vermeulen (90) successfully treated a patient with refractory CIDP, who had not achieved remission for 10 years, using HSCT. A large, single-center, open-label prospective study reported in 2020 (91) included 66 CIDP subjects with an average age of 43 years. The treatment-free remission rate at 6 months post-HSCT was 80%, while the rate of remission without adjunctive therapy exceeded 80% after one year, maintaining stability for up to five years. These findings indicate the potential of HSCT for long-term treatment of refractory CIDP. A meta-analysis conducted in 2023 (92) included 89 patients from 11 studies as of December 2022, with an average age of 42.1 years, and found a clinical improvement rate of 87% and an immunological drug-free remission rate of 81%. No HSCT-related deaths were reported, although some cases experienced adverse reactions such as infections and sepsis. The results of this meta-analysis support the efficacy and relative safety of HSCT for refractory CIDP, particularly in younger patients.

Post-HSCT treatment typically requires preventive measures against infections. The adverse reactions caused by immunosuppression after transplantation, such as viral and bacterial infections, fever, and acute liver function impairment, can largely be improved through appropriate treatments (93). Therefore, HSCT for refractory CIDP is generally safe and feasible. However, with the emergence of various novel therapies in the field of CIDP treatment, factors such as the complexity of the HSCT preconditioning process, the need for prolonged hospitalization, and the higher risk of early adverse reactions have impacted its consideration as a treatment option. Given that this therapy can provide significant efficacy and the possibility of long-term remission for patients who are resistant to conventional treatments, HSCT is currently only regarded as a last-resort treatment option for refractory CIDP.

#### 2.3.5 Complement inhibitors

Complement serves as a bridge between the innate and adaptive immune systems and plays a crucial role in various autoimmune diseases. In CIDP (94), antigen-antibody complexes activate the complement system via the classical pathway, forming the C1 complex (C1qrs), which subsequently activates C2 and C4, leading to the formation of C3 convertase. The MBL pathway can also participate in this process by cleaving C2 and C4, while the alternative pathway significantly amplifies the cleavage of C3. The membrane attack complex (MAC) generated from the final cleavage of C5 can directly damage Schwann cells and myelin, while fragments such as C5a (95) recruit and activate inflammatory cells, collectively leading to chronic, progressive nerve damage. Complement-targeted therapies have been successfully applied to many autoimmune antibody-mediated diseases (96), such as generalized myasthenia gravis (gMG). Studies indicate that nerve damage in certain subtypes of CIDP may be associated with complement activation (95, 97, 98). This suggests that the strategic application of complement inhibitors to precisely block key steps in complement activation may represent a promising therapeutic strategy.

SAR 445088 (BIVV 020) is a humanized monoclonal antibody that selectively inhibits the classical pathway of the complement system while preserving the immune functions of other complement pathways, allowing the body to maintain its defense against pathogens and thereby reducing side effects such as infections. The first human study conducted on 93 healthy individuals demonstrated good tolerance to SAR 445088, with no severe adverse reactions reported (95). Currently, an open-label, non-randomized, phase II trial (99) (NCT04658472) is underway to evaluate the efficacy, safety, and tolerability of SAR 445088 in 90 CIDP subjects, aiming to provide a basis for C1-targeted therapy in CIDP patients. Interim results indicate that 87% of standard of care (SOC) subjects improved or maintained stability after switching to SAR 445088, and 89% of SOC refractory participants showed improvement or stability after 24 weeks of SAR 445088 treatment, with controlled safety observed throughout the study. Three global phase III studies have been initiated to assess the efficacy and safety of SAR 445088 in adult patients with refractory CIDP (NCT06290128) and adult patients with CIDP receiving IVIG maintenance therapy (NCT06290141), as well as the long-term safety and efficacy of treating CIDP (NCT06859099).

Eculizumab is a humanized anti-C5 monoclonal antibody that specifically binds to the complement C5 protein, thereby inhibiting C5 cleavage and preventing the pro-inflammatory cascade effects of C5a and C5b-9 (100). Due to its significant suppression of complement activity, Eculizumab treatment may increase the risk of infections, particularly meningococcal infections. Thus, vaccination is required prior to administration, and long-term use should involve monitoring for adverse events. The efficacy and safety of Eculizumab in treating patients with Myasthenia Gravis (MG) and Neuromyelitis Optica Spectrum Disorder (NMOSD) have been validated (101, 102). Several small randomized controlled trials (RCTs) have investigated the potential for Eculizumab to improve disability levels in Guillain-Barré Syndrome (GBS) and its safety, but no significant conclusions have been reached, indicating the need for larger studies to confirm the clinical effects of Eculizumab (103). Currently, there are no experimental studies on Eculizumab in patients with CIDP. Ravulizumab has the same mechanism of action as Eculizumab but with a longer dosing interval, which can reduce the frequency of infusions, and it is considered to have the potential to replace Eculizumab (104). Although there are currently no applications related to peripheral neuropathy, its pharmacological properties indicate potential research value in the treatment of CIDP. Furthermore, as the first complement C2 drug to enter clinical stages, a phase III clinical study comparing the efficacy of empasiprubart and IVIG in treating CIDP patients (NCT06920004) has recently commenced. A Phase III clinical trial of the C1s inhibitor DNTH103 (NCT06858579) is currently underway. Based on the mechanism of action, other complement-related drugs such as GL-2045 and Zilucoplan (105) also have potential therapeutic effects, although they have not yet been tested in CIDP.

#### 2.3.6 Inhibitors of B cell signal pathway

Bruton tyrosine kinase (BTK) is a key kinase in the B cell receptor and Fc receptor signaling pathways (106). BTK inhibitors may exert therapeutic effects on CIDP by inhibiting B cell activation and proliferation, thereby reducing the production of autoantibodies and suppressing macrophage-mediated demyelination. Currently, BTK inhibitors have been used in malignant hematological diseases such as chronic lymphocytic leukaemia (CLL) and Waldenström macroglobulinemia (WM) (107), and they show potential for application in many autoimmune diseases (108). Castellani et al. reported improvements in three patients with anti-MAG neuropathy associated with WM after treatment with ibrutinib (109). Subsequently, a multicenter cohort study suggested that zanubrutinib might be effective in anti-MAG neuropathy (110). A Phase II clinical trial (NCT05939037) investigating zanubrutinib in combination with rituximab for the treatment of anti-MAG neuropathy is currently underway.

The B-cell-activating factor of the TNF family (BAFF) and A proliferation-inducing ligand (APRIL) are key cytokines for B cell survival and differentiation. Studies have shown that BAFF levels are significantly elevated in patients with CIDP (111), indicating that BAFF/APRIL may be involved in the pathogenesis of CIDP. Currently, there are several BAFF/APRIL inhibitors that have demonstrated good efficacy in autoimmune diseases such as SLE (112). Atacicept and Telitacicept are fully human recombinant fusion proteins that can simultaneously block BAFF and APRIL, while Belimumab and blisibimod are recombinant human IgG1 monoclonal antibodies that inhibit BAFF.

BTK inhibitors and BAFF/APRIL inhibitors suppress B cell activation or survival by targeting different aspects of the B cell pathway, showing therapeutic potential for CIDP. However, clinical studies on the use of these drugs for CIDP treatment have not yet been conducted, and their efficacy and safety require further validation.

## 3 Progress in the treatment of AN

AN differs from typical CIDP in clinical manifestations, pathological features, and treatment responses. Although it has previously been classified as a special subtype of CIDP and has followed the immunotherapy protocols of CIDP, its treatment strategies are unique. This section will focus on the unique treatment advancements for AN, without reiterating the treatment methods that are the same as those for typical CIDP. **Table 1** summarizes the current and potential treatment options for AN.

**Table 1:** Summary of potential treatments for Autoimmune Nodopathy.

Therapeutic Approach	Drug/Therapy	Target	Mechanism	Adverse Effects	Major Clinical Evidence in AN
B-cell targeted therapy	Rituximab	CD20	Depletes B cells, reducing autoantibody production	Infection(high-dose regimens),requires B-cell monitoring	A systematic evaluation (124) reported a 96% efficacy rate among 25 patients; A retrospective study involving 40 patients (125) showed a 77.3% efficacy rate; The RECIPE study (NCT03864185)
Traditional Immunotherapy	Corticosteroids	B Cells and Plasma Cells, Complement System, NF-κB Inflammatory Pathway, etc.	Rapid Suppression of Inflammatory Responses, Reduction of Pathogenic Antibody Production, Inhibition of Complement Pathways, etc.	Osteoporosis, electrolyte imbalance, hyperglycemia	A systematic review indicated efficacy in 40%-60% of patients (114)
	PE/IA	Antibodies in Circulation	Clearance of Pathogenic Antibodies in Circulation	Hypocalcemia, hypotension, allergic reactions, bleeding tendencies	PE: A systematic review (120) showed a 75% efficacy rate among 4 patients; IA: A case report (122), effective
	IVIG	Complement System, Pathogenic Autoantibodies, B Cells, etc.	Neutralization and Clearance of Autoantibodies, Inhibition of Complement Deposition and Activation, Inhibition of Autoantibody Production, etc.	Headache, fever, hypertension, thromboembolism	One study (117) indicated that only 20% of 25 patients with NF155 IgG4 antibodies showed a response;Several case reports (3, 119–121) demonstrated early responses with other antibody subtypes
FcRn Blockers	Efgartigimod	FcRn	Blocks FcRn to accelerate IgG degradation	Infection, headache, myalgia	In the ADHERE study, the Phase A response rate in AN was 77.8% (152); A prospective study involving 4 patients (153) reported a 100% efficacy rate
B-cell targeted therapy	Ofatumumab	CD20	More potent CD20 binding and prolonged B-cell depletion compared to rituximab	Infections, headache, injection-related reactions (lower immunogenicity than RTX)	A prospective study involving 8 patients (145), 100% effective
BAFF/APRIL inhibitors	Telitacicept	BAFF/ APRIL	Suppresses survival of plasma cells and mature B cells, reducing autoantibodies	Infections, rash, fever	A case report (149), effective
Plasma cell-targeted therapy	Daratumumab	CD38	Specifically targets long-lived plasma cells to reduce antibody production	Infusion-related reactions, upper respiratory infections, hematologic disorders	A case report (140), effective
Proteasome inhibitors	Bortezomib	Proteasome	Induces plasma cell apoptosis, inhibiting persistent autoantibody production	Heart failure, peripheral neuropathy	A case report (141), effective
Cell therapy	HSCT	Immune system	Rebuilds immune system and eliminates pathogenic B cells	Infection, fever, acute liver injury	Two case reports (150, 151), 100% effective
	CAR-T	CD19, BCMA	Eliminates autoantibody-producing B cells and plasma cells	ICANS, CRS	A case report (88), effective
Complement inhibitors	SAR 445088	Complement system	Blocks complement cascade activation	Infection	No clear evidence yet
B-cell targeted therapy	Inebilizumab	CD19	Targeting and eliminating CD19-positive B cells, reduces the production of IgG4-dominant autoantibodies	Urinary tract infections, joint pain	No clear evidence yet

ICANS Immune Effector Cell-Associated Neurotoxicity Syndrome, CRS Cytokine Release Syndrome.

### 3.1 Current treatments

Currently, the treatment for AN primarily relies on immunomodulation and targeted therapies, particularly rituximab, corticosteroids, and plasma exchange (PE). Among these, rituximab is the preferred treatment method for IgG4-positive patients, while IVIG is ineffective in the majority of AN patients (113).

#### 3.1.1 Corticosteroids

Glucocorticoids remain one of the most commonly used medications in the treatment of AN, with approximately 50% of AN patients responding well to glucocorticoids (114). Considering the pathogenesis of AN is related to autoantibodies, glucocorticoids can reduce B cell activity and decrease the production of pathogenic antibodies, thereby alleviating the attack on the Ranvier nodes. There may be differences in the response to glucocorticoids among AN patients with different antibody subtypes. The response rate to glucocorticoids in patients positive for anti-NF155 is approximately 51%, while the response rate in patients positive for anti-CNTN1 is about 73% (115). However, patients with anti-Caspr1 antibody positivity tend to have a poor response to glucocorticoids. It is noteworthy that while some AN patients respond to corticosteroids, monotherapy often fails to achieve sustained remission. In a study involving AN patients positive for anti-CNTN1 antibodies (116), the majority (63.6%) of patients receiving corticosteroid treatment showed a therapeutic response; however, three cases required subsequent treatment with rituximab, and two cases needed combined IVIg to achieve remission. Therefore, corticosteroids are still recommended as the initial treatment option for AN, and can be used in conjunction with or sequentially with other drugs to consolidate efficacy.

#### 3.1.2 IVIG

Although IVIG is the first-line treatment strategy for CIDP, its efficacy in patients with IgG4 subtype AN is suboptimal. Studies indicate that the treatment efficacy of IVIG for patients predominantly expressing IgG4 subtype is less than 20%, which is significantly lower than that for antibody-negative CIDP patients (117). This is related to the structural characteristics of IgG4. The half-antibody exchange property of IgG4 antibodies, along with the unique structural features of the hinge region, prevents them from participating in the complement activation pathway, and the Fc domain has a relatively low affinity for receptors on effector cells (6, 118). Since IVIG primarily exerts its effects by inhibiting complement deposition and activation, and binding to inhibitory Ig receptors (119), most paranodal antibodies (NF155, CNTN1, Caspr1) predominantly being IgG4 leads to a poor response to IVIG. However, patients with nodal antibodies (NF186/NF140) typically show a therapeutic response to IVIG (3), which is associated with a higher proportion of IgG1/3 antibodies (120) among them. These antibody subclasses can induce complement deposition and activation, making them responsive to IVIG treatment. Notably, some patients who are positive for pan-NF (NF-186/140/155) antibodies (3, 120, 121) initially respond to IVIG, but the efficacy decreases over time, potentially related to the shift of antibodies from IgG3 to IgG4 during disease progression. In a report by Appeltshauser et al. (119) involving three patients with CNTN1 IgG antibody positivity, A patient dominated by IgG3 antibodies showed significant improvement after the application of IVIG, while two other patients initially dominated by IgG3 antibodies later transitioned to IgG4 antibodies, resulting in a corresponding decrease in efficacy. This study further confirms that IgG3 is a predictive factor for the response to IVIG therapy, with a higher proportion of IgG3 generally correlating with a better response to IVIG treatment. Therefore, measuring the proportions of the patient’s own antibody subclasses may serve as a potential indicator for predicting the response to IVIG therapy, although this may be challenging in clinical practice and warrants further investigation.

#### 3.1.3 Plasma exchange

Plasma exchange is commonly used in critical conditions or when there is a poor response to IVIG or glucocorticoids, and it is also employed as a bridging therapy prior to the use of immunosuppressive agents. In a study by Burnor et al. (120) involving five patients with anti-NF155 positive antibodies, one patient responded effectively to high-dose glucocorticoid treatment, while three out of the remaining four patients showed improvement following plasma exchange. This suggests that patients with anti-NF155 positive antibodies may respond better to plasma exchange than to glucocorticoid therapy. Based on clinical experience, a combination of plasma exchange and B cell depletion therapy may yield a more rapid and robust effect. However, the efficacy and safety of this combined treatment warrant further investigation through larger-scale clinical trials. It is worth noting that for maintenance therapy, the tolerance of plasma exchange may be lower than that of IVIG and corticosteroids, or it may lead to more safety concerns.

Immunoadsorption therapy can selectively remove circulating IgG antibodies through specific adsorption, thereby reducing immune attacks on the Ranvier’s node region and improving nerve conduction function. It is an effective alternative to plasma exchange. For patients with rituximab-resistant AN, IA may rapidly decrease antibody titers in the short term. However, long-term control of AN still requires immunomodulatory therapy. Liu et al. reported a case of a patient who was positive for anti-Caspr1 and allergic to RTX (122), presenting with loss of proprioception, ataxia, coarse tremors, and distal limb weakness. After three sessions of IA treatment, the patient’s condition significantly improved. The combined use of rituximab and immunoadsorption may also provide additional benefits, but further research is needed to confirm this.

#### 3.1.4 Rituximab

The autoimmune nature of AN suggests that B cell depletion therapy may benefit these patients. Rituximab treatment can effectively reduce the levels of IgG4 autoantibodies (123). For patients with IgG4 positivity exhibiting refractory characteristics to IVIG, rituximab is recommended as the preferred treatment of choice. A meta-analysis included 25 IgG4-positive AN patients who had poor responses to first-line treatment, with 24 patients (96%) demonstrating a good response to rituximab, which is superior to the response rate in the overall CIDP population (75%). Additionally, 17 patients (68.0%) showed a significant decrease in autoantibody levels post-treatment, indicating a positive impact of rituximab on IgG4-positive patients (124). In a study (125), 40 patients positive for anti-NF155 antibodies received rituximab therapy after inadequate responses to other treatments, with 77.3% of patients exhibiting good responses. Some small case reports have also demonstrated the efficacy of RTX in other IgG4 subtypes (126, 127).Thus, rituximab can be considered an effective treatment option for refractory AN. The RECIPE study in Japan (NCT03864185) includes 5 patients who are positive for IgG4 antibodies and 10 patients who are antibody-negative, aiming to evaluate the efficacy and safety of rituximab in refractory IgG4 antibody-positive or negative patients, with results yet to be published. However, some studies have indicated that the efficacy of rituximab diminishes with the progression of the disease and the extent of axonal damage (128). In a pediatric study (129), among six patients positive for NF155 antibodies, two children experienced minimal symptom improvement with rituximab treatment, which may be related to the fact that their disease duration exceeded six months at the time of treatment. Therefore, for patients with a clear diagnosis of AN who do not respond to first-line treatments, rituximab should be administered as early as possible.

In recent years, there has been an increasing amount of research on the use of low or moderate doses of rituximab in the treatment of AN. As previously mentioned, the commonly used high-dose rituximab treatment regimens carry risks of infection and reduced lymphocyte counts, and the convenience and cost of frequent treatments are worth considering. The application of low-dose rituximab regimens in autoimmune diseases such as NMOSD and AE has shown favorable results (130). Therefore, exploring the efficacy and safety of low or moderate dosing regimens for AN is of significant importance. A prospective observational study (131) included 19 AN patients who received low-dose rituximab via fractionated intravenous infusion (100 mg on the first day and 500 mg on the second day), with treatments repeated every six months. The results indicated that 94.7% (18/19) of patients experienced significant improvements in neurological function scores and successfully discontinued oral medications, with no infusion-related adverse reactions reported. Another case report (132) describes a patient positive for anti-NF155 IgG4 antibodies, who showed continuous clinical improvement and a significant reduction in antibody titers after receiving two 500mg rituximab injections spaced 6 months apart. This low-dose regimen provides both efficacy and safety, potentially shortening hospitalization times and offering an effective treatment option for certain patients. Due to the small sample size of the current studies, larger clinical trials are needed to confirm these findings.

### 3.2 Potential therapeutic strategies

Patients with AN exhibit a lower response to first-line treatment compared to those with CIDP, and the treatment process is complex and prolonged. In clinical practice, it is often necessary to use a combination of multiple medications or add immunosuppressants such as mycophenolate mofetil, azathioprine, and cyclosporine for long-term maintenance. Moreover, the use of rituximab is limited by its high cost and convenience, and it is difficult to treat some patients. Therefore, exploring new treatment strategies is essential. As an antibody-mediated autoimmune disease, potential therapeutic directions of AN mainly include pathogenic antibody removal, B cell/plasma cell targeted therapy, and complement inhibition. **Table 1** provides a summary of potential treatment options for AN.

#### 3.2.1 Plasma cell targeted therapy

Research on the source of autoantibodies in AN patients indicates that, although most patients’ autoantibodies originate from memory B cells and short-lived plasma cells/plasmablasts, a small subset of patients may have antibodies derived from long-lived plasma cells (133). Under persistent antigenic stimulation from chronic infections, a small subset of autoreactive plasma cells eventually differentiate into long-lived plasma cells (134), which reside in the bone marrow or inflammatory sites and maintain long-term survival. During the development of AN, these cells may continuously produce pathogenic IgG4 antibodies, thereby increasing the risk of disease relapse. Since these cells do not express the CD20 antigen, they exhibit poor responses to therapies targeting B cells, such as rituximab, and are also difficult to eliminate with conventional immunosuppressants. Therefore, directly targeting long-lived plasma cells with Daratumumab and proteasome inhibitors may represent a more effective treatment option for these patients.

Daratumumab is a humanized monoclonal antibody targeting CD38, specifically acting on long-lived plasma cells to reduce antibody production. It has been approved for the treatment of multiple myeloma (135) and has also shown promising therapeutic effects in autoimmune hemolytic anemia (136), systemic lupus erythematosus (137), and autoimmune encephalitis (138, 139). However, there is currently only one retrospective case series study that addresses the application of this drug in in antibody-mediated neurological diseases (140). This study described a critically ill AN patient with SLONM and multiple myeloma, who was switched to daratumumab treatment after conventional therapies, including glucocorticoids, plasma exchange, and rituximab, proved ineffective. The results indicated that the patient’s clinical symptoms were alleviated, and antibody levels significantly decreased. Nonetheless, the safety concerns associated with this drug cannot be overlooked. Among seven patients with antibody-mediated refractory autoimmune neuropathy who received treatment, five experienced severe adverse reactions, one of which even resulted in death. Consequently, Daratumumab has demonstrated certain therapeutic potential, but larger-scale studies are needed to explore its efficacy and safety.

The proteasome inhibitor bortezomib inhibits the sustained production of autoantibodies by promoting plasma cell apoptosis and also has a clearing effect on long-lived plasma cells. This mechanism makes it a potential alternative for AN patients who have failed rituximab treatment. A report on a patient positive for anti-pan-NF antibodies (141) showed the therapeutic effect of proteasome inhibitors. This patient, who experienced gradual deterioration of symptoms despite treatment with IVIG, PE/IA, and corticosteroids, did not improve with RTX therapy. However, after 2 weeks of treatment with Bortezomib, clinical symptoms improved, and subsequent NCS also showed improvement. Larger clinical trials are hoped to further validate the efficacy of Bortezomib in AN.

#### 3.2.2 Other B cell targeted therapies

For a small subset of patients who respond poorly to Rituximab, more effective anti-CD20 treatment may offer a better option. Ofatumumab (OFA), as the first fully human anti-CD20 monoclonal antibody (142), has different epitopes compared to Rituximab and can bind more tightly to CD20+ B cells (143), achieving equivalent efficacy at lower doses (144). A recent open-label, prospective study (145) demonstrated that after 24 weeks of treatment with OFA (20 mg subcutaneous injection weekly, changing to once a month after 1 month) in 8 patients with AN, all achieved clinical improvement without any allergic reactions or serious adverse events. These results suggest that OFA may provide an effective alternative therapy for patients who do not respond to RTX, but larger-scale studies are still needed to validate its efficacy and safety.

Inebilizumab is a humanized anti-CD19 monoclonal antibody that targets and depletes CD19-positive B cells through antibody-dependent cellular cytotoxicity (146). The MITIGATE study (NCT04540497) demonstrated (147) that this drug significantly reduces the risk of relapse in patients with IgG4-related disease (IgG4-RD) and can rapidly and sustainably deplete peripheral B cells. Consequently, it is received FDA approval in the United States for the treatment of IgG4-RD by 2025. Although there are currently no studies on this drug for AN, its mechanism of action and efficacy in treating IgG4-RD suggest it may have therapeutic potential for AN patients who are positive for IgG antibody subtypes.

#### 3.2.3 BAFF/APRIL targeted therapy — Telitacicept

Telitacicept is a dual inhibitor targeting BLyS and APRIL, which effectively suppresses the survival of plasma cells and mature B cells (148). Its efficacy and safety have been confirmed in diseases such as systemic lupus erythematosus (SLE) and rheumatoid arthritis (RA). In 2023, a case was reported involving an anti-NF155 positive AN patient who was resistant to conventional treatments, including plasma exchange and rituximab (149). After treatment with Telitacicept, clinical symptoms improved, as evidenced by the INCAT and I-RODS scales, and the anti-NF155 antibody titers consistently decreased. This case suggests that Telitacicept may be a potential new therapy for anti-NF155 positive AN, particularly for patients with high antibody titers or those who are refractory to treatment, although there are currently no clinical trial studies on Telitacicept for AN.

#### 3.2.4 Hematopoietic stem cell transplantation

Hematopoietic stem cell transplantation (HSCT) has shown potential in the treatment of AN by reconstructing the immune system to eliminate pathogenic B cells. In 2016, Scheibe et al. (150) reported a case of a patient with AN who underwent autologous hematopoietic stem cell transplantation. Post-surgery, the patient experienced a significant reduction in clinical symptoms, with NF155 antibodies decreasing by more than half, and there was no recurrence during a five-year follow-up. In 2024, Afanasiev et al. (151) reported the first case of a patient with anti-Caspr1 antibodies who was unresponsive to rituximab and underwent HSCT treatment 30 months after the onset of the disease. Three months post-treatment, the anti-Caspr1 antibodies became negative, and the patient’s daily living abilities and muscle strength gradually improved. The significant benefits of HSCT require further clinical trial validation. However, the rarity of this disease, along with the complexity of HSCT applications and the risk of adverse reactions, limits its viability as a treatment option and the feasibility of larger-scale studies.

#### 3.2.5 FcRn blockers — efgartigimod

As an FcRn blocker, efgartigimod can lower IgG levels by inhibiting FcRn and is currently used for CIDP and many IgG-mediated autoimmune diseases. Given that the related antibodies in AN are primarily of the IgG4 subtype, efgartigimod shows promising prospects for treating AN. At the 2025 PNS conference, the ADHERE study (152) reported that 4% of patients who underwent antibody testing were AN patients. Efgartigimod significantly reduced their IgG antibody levels, with a phase A response rate of 77.8%, higher than the overall CIDP response rate of 66.5%. This preliminary data demonstrates the efficacy of efgartigimod in AN. In a recent prospective study involving four patients (153), efgartigimod treatment showed rapid efficacy within two weeks, consistent with the ADHERE trial. Furthermore, the notable effectiveness for IgG3/NF186 cases also underscores the significance of exploring personalized treatment strategies targeting IgG subclasses and antigen types.

#### 3.2.6 Other treatments

CAR-T therapy has shown promise in eliminating pathogenic B cells and plasma cells, particularly in refractory CIDP. Recently, J. Motte et al. (88) reported on two patients undergoing anti-CD19 CAR-T therapy, one of whom was a patient with anti-NF-155 IgG antibody-positive AN who had a poor response to IVIG, PE, and RTX treatment. Notably, after four months of CAR-T therapy, there was a significant improvement in motor function, and the anti-NF-155 IgG antibody turned negative, indicating the potential of CAR-T therapy in treating refractory AN.

Despite AN antibodies being predominantly IgG4, studies have indicated that pan-NF(NF-140/186/155) (98, 121, 154) and CNTN-1 (119) IgG3 subtypes are associated with complement binding and activation, suggesting that complement inhibitors may be a treatment option. These drugs have demonstrated significant efficacy in other complement-related diseases, but their safety and effectiveness in AN still require further research.

In addition, considering the pathogenesis of AN related to Treg dysregulation and demyelination, the regulation of the Treg pathway (155) and strategies to promote Schwann cell repair and myelin regeneration are also being gradually explored, although further experimental results are needed to support these efforts.

## 4 Conclusion

CIDP has an incompletely understood pathogenesis, and clinical treatment continues to face numerous challenges. The current first-line treatment options include corticosteroids, IVIG, and plasma exchange. For cases of diseases with poor efficacy or refractory conditions, it may be necessary to consider combination therapy with immunosuppressants or to explore novel drugs and multi-target treatments. Recently, subcutaneous efgartigimod has been approved for CIDP, marking the beginning of a new treatment area suitable for patients who are insufficiently responsive to existing first-line therapies, intolerant, or in need of a convenient alternative. As clinical evidence continues to accumulate, its future therapeutic positioning may be adjusted.

Notably, autoimmune neuropathy, which was previously classified as a refractory subtype of CIDP, has now been identified as a distinct disease. Due to its unique pathological mechanisms and treatment responses, the therapeutic strategies for AN differ slightly from those for CIDP, with rituximab recommended as the preferred agent for AN. Corticosteroids and plasma exchange are primarily used for acute symptom control, while IVIG is generally ineffective in most AN cases. For refractory cases or during the maintenance treatment phase, the use of immunosuppressive agents in combination may be considered. Some potential novel drugs show promising application prospects. However, due to the low incidence of AN, current studies are limited, and further research is anticipated to demonstrate their therapeutic value.

The differences in treatment responses between AN and CIDP also suggest that for CIDP patients who clinically present with tremors, cranial nerve involvement, and sensory ataxia, along with significantly elevated cerebrospinal fluid protein and poor response to IVIG, it is of great importance to conduct testing for nodal and paranodal antibodies. The association between different antibody subtypes and treatment responses warrants further investigation to facilitate the development of personalized treatment.
